# Genomic epidemiology of mecC-carrying Staphylococcus aureus isolates from human clinical cases in New Zealand

**DOI:** 10.1099/acmi.0.000849.v2

**Published:** 2024-09-05

**Authors:** Hilary Miller, Julia Howard, Juliet Elvy, Patrick Campbell, Trevor Anderson, Sarah Bakker, Alexandra Eustace, Hermes Perez, David Winter, Kristin Dyet

**Affiliations:** 1 Institute of Environmental Science and Research, Wellington, New Zealand; 2 Microbiology Department, Canterbury Health Laboratories, Christchurch, New Zealand; 3 Department of Microbiology, Awanui Labs, Dunedin, New Zealand; 4 Infection Management Service, Christchurch Hospital, Christchurch, New Zealand

**Keywords:** antimicrobial resistance, *mecC*, MRSA, One Health, *Staphylococcus aureus*, whole-genome sequencing

## Abstract

In 2011, a novel methicillin resistance gene, *mecC*, was described in human and bovine *Staphylococcus aureus* isolates. *mecC-*positive *S. aureus* is most commonly associated with livestock and wildlife populations across Europe and is particularly prevalent in hedgehogs, but only occasionally causes human infections. In this study, we characterize and investigate the origin of two human *S. aureus* isolates containing *mecC* genes from New Zealand. The two isolates were identified from patients with severe invasion infections as part of an *S. aureus* bacteraemia study. Whole-genome sequencing was used to characterize staphylococcal cassette chromosome *mec* (SCC*mec*) elements and perform phylogenetic comparisons with publicly available strains from *mecC*-associated clonal complexes, including isolates from hedgehogs from New Zealand and Europe/United Kingdom (UK), and livestock, wildlife and human isolates from Europe/UK. The two isolates from our study have almost identical SCC*mec* type XI elements containing a *mecC* gene. However, this gene contains a premature stop codon, consistent with the methicillin-susceptible phenotype observed for these isolates. Core genome SNP analyses showed that the two isolates are 234 SNPs apart and are most closely related to an isolate obtained from a New Zealand hedgehog. However, there are considerable differences in the *mecC* mobile element between the human and hedgehog isolates, indicating the presence of an as-yet-unknown reservoir of *mecC S. aureus* in the New Zealand environment.

## Data Summary

Isolates sequenced in this study have been submitted to the National Center for Biotechnology Information under BioProject accession number PRJNA1029301. Accession numbers for other samples included in the study are given in Supplementary Material, available in the online version of this article.

**Impact Statement**

Methicillin-resistant *Staphylococcus aureus* (MRSA) poses a significant public health threat worldwide. Methicillin resistance in humans is commonly mediated by the *mecA* gene. However, in 2011, a distinct methicillin resistance gene, *mecC* was identified. *mecC-*carrying *S. aureus* has now been found in diverse livestock and wildlife populations and represents an emerging source of MRSA cases in humans but is rarely reported outside of Europe. Our study is the first report of *S. aureus* isolates with a *mecC*-containing staphylococcal cassette chromosome *mec* type XI element from any species in New Zealand, thus expanding the geographic range where *mecC S. aureus* is known to occur. Through whole-genome sequencing and phylogenetic comparison with publicly available isolates, we show that these isolates form a clade with isolates from a New Zealand hedgehog but have considerable differences in their *mecC* mobile elements, indicating there may be another source of *mecC* in the New Zealand environment. Our study shows the need for further sampling of *S. aureus* from livestock, wildlife and humans in New Zealand to ascertain possible public health risks from *mecC-*carrying *S. aureus*.

## Introduction

Methicillin-resistant *Staphylococcus aureus* (MRSA) is a major global healthcare issue. Methicillin resistance was originally associated with the acquisition of the *mecA* gene, which encodes an altered penicillin-binding protein 2a [1]. The *mecA* gene is located on a mobile genetic element, staphylococcal chromosomal cassette *mec* (SCC*mec*), which is incorporated into the *S. aureus* chromosome [2]. In 2011, a homologue of *mecA*, named *mecC*, was discovered in human and bovine *S. aureus* populations in the United Kingdom (UK) and Denmark [3]. The *mecC* gene was found to be on a novel SCC*mec* element, named type XI, which is most commonly found in clonal complexes 130, 425, 599 and 1943 [4].

Since 2011, *mecC*-positive *S. aureus* isolates have been found in humans, livestock, companion animals and wildlife throughout Europe and the UK [4, 5,7]. Reports from outside of Europe are less common, but it has also been detected in domestic cats in Australia [8] and dairy cattle in Malaysia [9] and Brazil [10]. Detections of *mecC*-MRSA are particularly common in hedgehog populations across Europe, with prevalence as high as 60% found in populations in Sweden, Denmark and the Netherlands [11,13]. A recent study by Larsen *et al*. [14] found that *mecC*-MRSA in hedgehogs originated prior to the development of antibiotics and is likely related to colonization of hedgehogs with the penicillin-producing fungus *Trichophyton erinacei* rather than the widespread use of antibiotics in livestock farming.

Clinical infections of *mecC*-MRSA are uncommon in humans, but they can cause severe disease [15]. Surveillance of MRSA in Europe detected *mecC* in less than 1% of all MRSA [15], although slightly higher rates (around 2%) were reported in Denmark [16]. Although hedgehogs are a natural reservoir for S. *aureus*, and some *mecC*-MRSA strains from humans are closely related to those from hedgehogs [13], direct transmission between hedgehogs and humans has not yet been confirmed. However, potential transmission between humans and livestock animals has been detected in a small number of cases [7,17, 18].

Here, we characterize the genomes of two isolates of *S. aureus* with non-functional *mecC* genes from human clinical cases where severe invasion infection was present. Prior to this study, *mecC*-carrying *S. aureus* had not previously been detected in human clinical isolates in New Zealand (NZ). However, *mecC* has been detected in a European hedgehog in NZ, with 1 out of 17 hedgehogs sampled by Larsen *et al*. (2022) testing positive for this strain. In this study, we aim to investigate the origin of the human clinical isolates by comparing them with *mecC*-positive *S. aureus* isolates from hedgehogs, humans and other livestock.

## Methods

### Sample collection and culture

Blood cultures were obtained from two patients admitted to Christchurch Hospital (patient 1) and Dunedin Hospital (patient 2) with severe invasive infection/multifocal bacteraemia. Samples were incubated in a BD BACTEC^TM^ FX blood culture analyser at Canterbury Health Laboratory (patient 1) or Awanui Labs Dunedin (patient 2). Positive cultures were plated on to blood and chocolate agar (Fort Richard laboratories, NZ) and incubated at 35–37 °C, and the presence of *S. aureus* was confirmed by matrix-assisted laser desorption/ionization-time of flight (Bruker Daltonics, Germany). Direct disc diffusion susceptibilities and BD Phoenix^TM^ (Beckton Dickinson) automated broth microdilution were performed with susceptibilities interpreted using European Committee on Antimicrobial Susceptibility Testing breakpoint tables version 11.0 (https://www.eucast.org/ast_of_bacteria/previous_versions_of_documents). Isolates were submitted from the respective laboratories to ESR on nutrient agar slopes.

### Preparation of PCR DNA template

Boiled lysis cell suspensions were used as a source of DNA templates in all PCRs. Plate cultures were used to prepare cell suspensions, approximately equivalent to a 0.5 McFarland standard, in DNase/RNase-free water. Suspensions were heated at 99 °C for 20 min. The heated suspensions were centrifuged to pellet cellular debris and then stored at 4 °C. The neat supernatants were used as a source of DNA templates.

### PCR and *spa* typing

A real-time PCR assay was used to determine the presence of *mecA, mecC*, the *S. aureus* species-specific thermostable nuclease gene *nuc* and one of the two genes encoding Panton–Valentine leukocidin [19]. The polymorphic X region of the staphylococcal protein A gene (*spa*) was amplified as previously described [20]. PCR products were submitted to the sequencing laboratory at ESR for Sanger sequencing using an ABI 3130XL Sequencer. The *spa* sequences were analysed using Ridom StaphType software version 2.2.1 (Ridom GmbH, Würzburg, Germany). Sequences were automatically assigned repeats and *spa* types using the software.

### Whole-genome sequencing and assembly

Whole-genome sequencing (WGS) was performed using Oxford Nanopore and Illumina sequencing. DNA was extracted from culture using the DNeasy Blood and Tissue Kit (QIAGEN, Hilden, Germany) and prepared for Illumina sequencing using the Nextera XT Library Kit (isolate 1) (Illumina, San Diego, CA, USA) or the plexWell 384 Library Kit (isolate 2) (seqWell Beverly, MA, USA). Paired-end 150-bp sequencing was performed on the NextSeq 550 platform (Illumina, San Diego, CA, USA). Sequence quality checks, *de novo* assembly and species identification were performed using an in-house pipeline comprising fastp v. 0.20.1 [21], Centrifuge v. 1.0.4 [22], SKESA v. 2.3.0 [23] and QUAST v. 5.0.2 [24].

Nanopore sequencing was performed using the rapid barcoding kit (SQK-RBK004, Oxford, UK) run on a GridION flow cell FLO-MIN106 (R9.4.1). Reads were base called using Guppy v 6.1.1 (Oxford Nanopore Technologies 2023; https://nanoporetech.com) using the super-accurate base calling model (dna_r9.4.1_450bps_sup). Quality trimming was performed using Filtlong v. 0.2.1 (https://github.com/rrwick/Filtlong), removing reads less than 1000-bp and retaining 90% of the best reads. Nanopore reads were assembled using Flye v. 2.9 [25] using the default settings and then polished with Illumina data using Pilon v. 1.23.0 [26]. The genome was then circularized using Circlator v. 1.5.5 [27] and annotated using Bakta v. 1.5.0 [28].

MLST was performed by comparing Illumina-assembled genomes to the *S. aureus* PubMLST typing scheme [29] using MLST v 2.19.0 (https://github.com/tseemann/mlst). The whole genome average nucleotide identity was computed using FastANI v. 1.33 [30]. Antimicrobial resistance (AMR) and virulence genes were detected from the WGS data using AMRFinderPlus v. 3.11.4 with database version 2023-09-26.1 and default settings [31].

### Characterization of the SCC*mec* cassette

The SCC*mec* cassette was identified from the Bakta-generated annotations and by using the Annotate from Database tool in Geneious Prime v. 2022.2 (https://www.geneious.com). The reference genome LGA251 (GenBank ID: FR821779 [3]) was used as the source for Annotate from Database. The *mecC* coding sequence was translated and aligned to the reference using the Geneious aligner. SCC*mec* typing was performed using SCCmecFinder v. 1.2 [32].

### Phylogenetic analyses

Core genome SNP analyses were performed using Snippy v. 4.6.0 (https://github.com/tseemann/snippy). Phylogenetic trees were built from the core genome SNP alignments with IQtree [33] using the built-in model selection and 1000 bootstrap replicates.

### Comparison of SCC*mec* regions and *mecC* genes

The isolates used in the phylogenetic analyses which contained an SCC*mec* cassette were *de novo* assembled using Skesa v. 2.3.0 [23] using the default settings. The Geneious Prime ‘Annotate from Database’ operation was then used to annotate the SCC*mec* element in these isolates, using the SCC*mec* element from *S. aureus* LGA251 (GenBank ID: FR821779) as the source database. SCC*mec* elements were then extracted from the *de novo* assembly, aligned with MAFFT, and a phylogeny was built using RAxML v.8.2.11 [34], using the ‘rapid bootstrapping and search for best-scoring ML tree’ option with the GTR + GAMMA model and 500 bootstrap replicates.

For genomes from the NZ hedgehog isolates, where SCC*mec* was not identified using Geneious, a search for components of the SCC*mec* element was undertaken using a local blast search for the LGA251 SCC*mec* element. A blast search of the National Center for Biotechnology Information nucleotide database was also performed on the *mec*C gene from both the human clinical and hedgehog isolates from NZ.

## Results

Two isolates submitted to ESR as part of the national *S. aureus* bacteraemia surveillance programme were *mecC*-positive by PCR. Isolate 1 was submitted to ESR in January 2022, and Isolate 2 was submitted in December 2022. Both isolates were reported to be methicillin-susceptible by standard antimicrobial susceptibility testing performed at the referring laboratories using cefoxitin disc testing and oxacillin MIC according to the European Committee on Antimicrobial Susceptibility Testing clinical breakpoints (https://www.eucast.org/ast_of_bacteria/). The presence of a truncated *mecC* gene was subsequently confirmed by WGS, as described below. Isolate 1 was spa type t208 and isolate 2 was spa type t14284. These spa types differ by one repeat insertion.

A complete genome sequence for isolate 1 was obtained by the assembly of nanopore reads, followed by polishing with Illumina reads. The chromosome is 2 780 834 bp long and has a GC content of 32.9%. No plasmids were present in the assembly. Annotation by Bakta identified 2541 coding sequences, 61 tRNAs, 19 rRNAs and 85 non-coding RNAs (ncRNAs). The MLST was unique, so the genome was submitted to PubMLST and assigned to ST-7767.

**Fig. 1.**
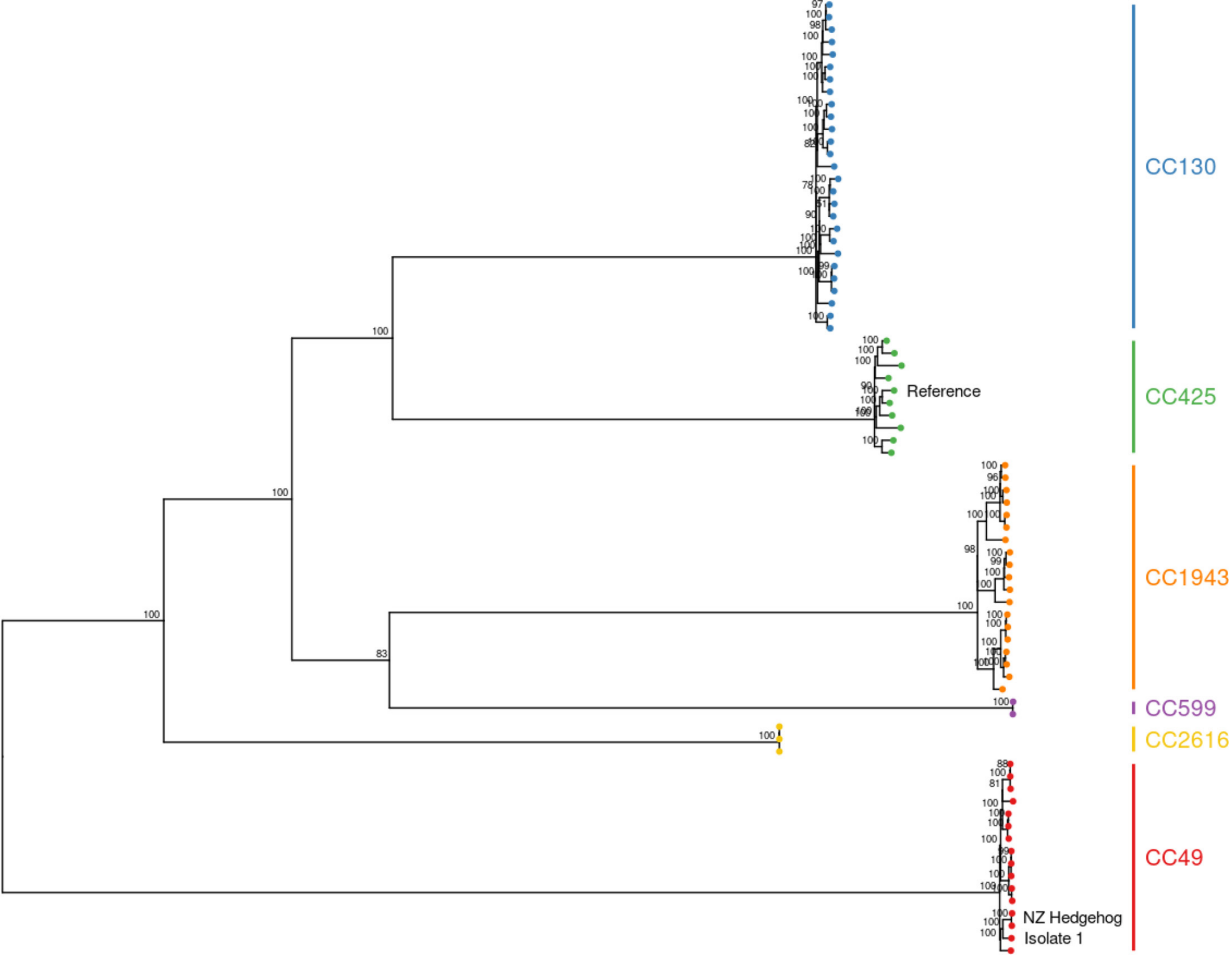
Comparison of isolate 1 with strains from clonal complexes previously associated with *mecC* (CC1943, CC599, CC2616, CC130 and CC425). The maximum likelihood tree is built from a core genome SNP alignment of 41 661 SNPs and is midpoint-rooted. Isolate 1 and the isolates from the NZ hedgehog are labelled.

Because isolate 1 was a unique ST and was not closely related to any other isolates in the ESR surveillance dataset at the time it was sequenced early in 2022 (unpublished data), we compared it with 75 isolates from clonal complexes commonly associated with *mecC* (CC49, CC130, CC425, CC599, CC1943 and CC2616) published by Larsen *et al*. in 2022. These isolates were chosen to represent the breadth of CC, ST and SCCmec variant types from that study (see Supplementary Material). The core genome alignment for these isolates included 41 661 variable sites, with the *S. aureus* LGA251 (GenBank ID: FR821779) genome used as the reference, and the model selection procedure identified TVMe + ASC + G4 as the best-fitting model of nucleotide substitution. The phylogenetic tree built from the alignment of core genome SNPs shows that isolate 1 is within the CC49 clade and is most closely related to two isolates sampled from the NZ hedgehog, ERR5417136 and ERR5417137 (Fig. 1).

A draft genome for isolate 2 was obtained from Illumina sequencing. The assembly comprised 46 contigs with N50 of 129584 bp. Annotation by Bakta identified 2445 coding sequences, 58 tRNAs, 5 rRNAs and 73 ncRNAs. This isolate was assigned to ST49 (CC49) by MLST and differs from isolate 1 at only one MLST locus. The whole genome average nucleotide identity between isolates 1 and 2 was 99.95%.

**Fig. 2.**
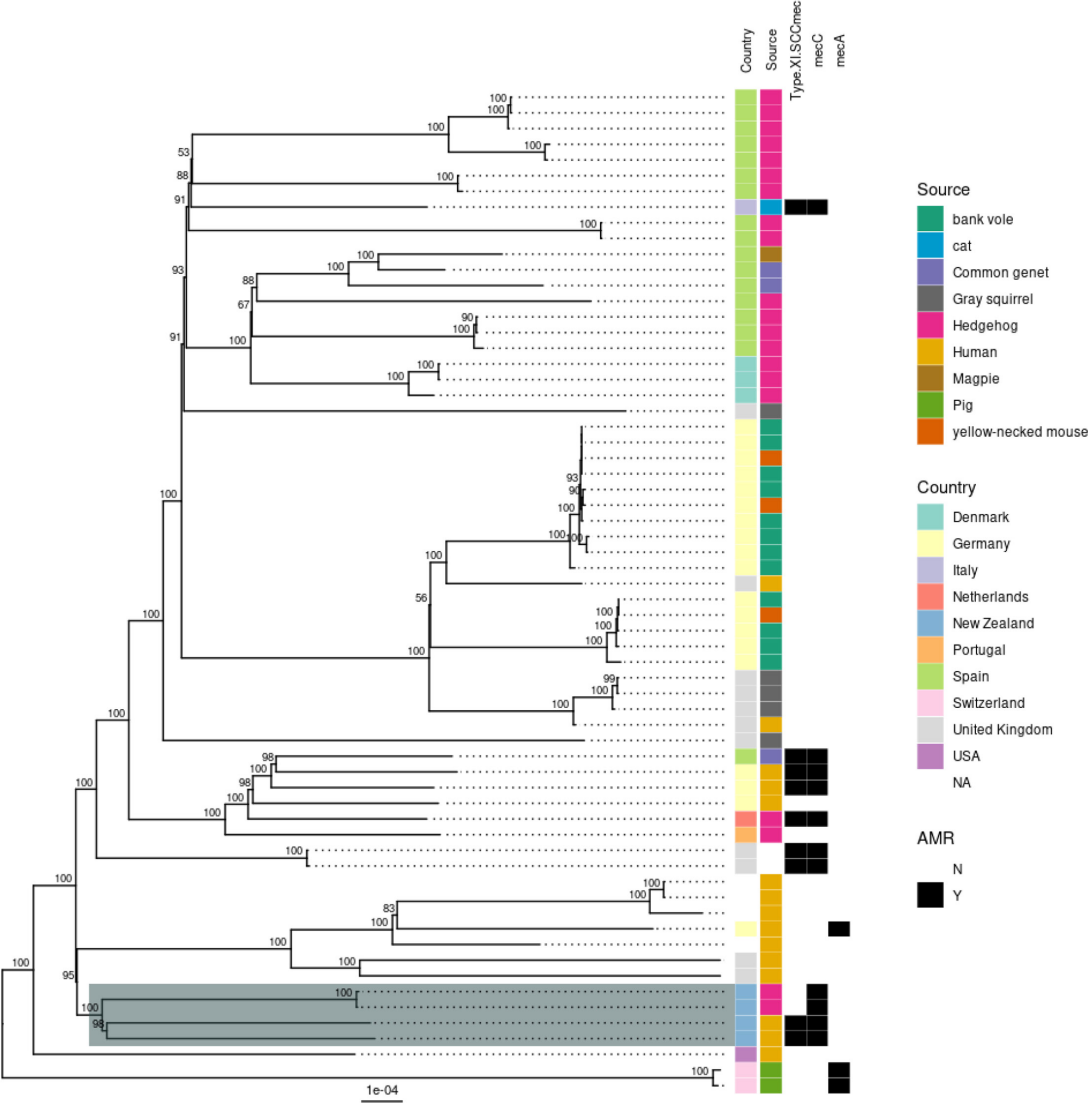
Maximum likelihood phylogenetic tree of 63 *S. aureus* CC49 isolates, based on 4273 core genome SNPs. The tree is mid-point-rooted. The two genomes sequenced in this study and the isolates from NZ hedgehogs are shaded grey. The country of origin, host species and the presence or absence of *mecA*, *mecC* and a full typeXI(8E) SCCmec element are shown in the coloured bars.

To further investigate the origin of our human isolates, we compared them to 61 other CC49 isolates, including 20 hedgehog isolates from NZ, Denmark, Portugal, Spain and the Netherlands (Larsen *et al*. (2022) [13, 35]), 12 human isolates from Germany and the UK (https://pathogen.watch/, accessed 14 November 2023) and 29 isolates from other European wildlife and livestock species [35,36] (https://pathogen.watch/, see Supplementary Material). This phylogeny is based on 4273 core genome SNPs, using the Tager 104 complete genome (GenBank ID: CP012409) as the reference and the TVMe + ASC model of nucleotide substitution. Tager 104 is an ancestral ST49 strain that was originally isolated in 1947 [37,38]. This analysis confirms that the two isolates sequenced in this study are most closely related to the two isolates from a NZ hedgehog (Fig. 2). On this tree, the NZ isolates, both hedgehog and human, form a separate clade from all European/UK isolates, which broadly cluster by country and host species. In this core genome SNP comparison, isolates 1 and 2 are 234 SNPs apart and 233–234 SNPs distant from the NZ hedgehog isolates.

**Fig. 3.**
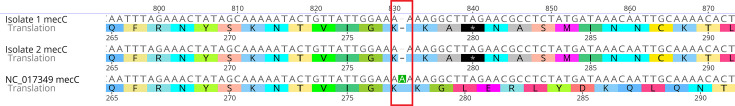
Alignment of *mecC* genes from isolates 1 and 2 and the reference sequence NC_017349. Only the region containing the first frameshift mutation (highlighted in the red box) and the subsequent premature stop codon (denoted by the asterisk in the amino acid sequence) are shown.

The *mecC* gene was identified by PCR in both isolates, and analysis of the annotated genome assemblies showed a full SCC*mec* element was present. This was identified by SCCmecFinder as a type XI(8E) SCC*mec* element. Pairwise nucleotide identity between isolates 1 and 2 across this region is 99.99%. The *mecC* genes from the two isolates are identical and contain single base pair deletions at positions 831 and 1012, which introduce a premature stop codon and likely render the gene non-functional (Fig. 3). These deletions were also found in the Sanger sequencing data and are consistent with the methicillin-susceptible phenotype observed for these isolates. A blast search of the GenBank Nucleotide database did not find any other *mecC* genes containing similar deletions.

The SCC*mec* elements from our isolates are most closely related to SCC*mec* elements from isolates in clonal complexes 130, 425 and 1943 (Fig. S1). SCC*mec* elements do not cluster by clonal complex, and only 7 out of 61 other CC49 isolates have a full SCC*mec* type XI element. The two NZ hedgehog isolates have a partial SCC*mec* element including a functional *mecC* gene. However, a blast search revealed the NZ hedgehog *mecC* gene has 100% identity with *mecC* allotype C2 from *Mammaliicoccus sciuri* (formerly *Staphylococcus sciuri*) and has only approximately 96% identity with *mecC* from the other isolates in our study, including the two human isolates we sequenced.

Analysis of AMR and virulence genes using AMRFinder found the beta-lactamase *blaZ* and the tetracycline resistance gene *tet* [37] in both isolates. The leukocidin-encoding *lukE* gene was detected in genomic data in both isolates. Neither isolate contained Panton–Valentine leucocidin genes (tested with both rtPCR and WGS) or the immune-evasion genes *chp, sak* and *scn* [39]. A full list of virulence genes identified is listed in Table S2 .

## Discussion

The *mecC*-PCR-positive *S. aureus* isolates reported in this study are, to our knowledge, the first human clinical cases of *mecC S. aureus* from NZ. National MRSA surveillance studies conducted by ESR have been screened for *mecC* by PCR since 2014. However, prior to the national *S. aureus* bacteraemia surveillance programme, which ran from January 2021 to December 2023, no systematic molecular surveillance of methicillin-susceptible *S. aureus* (MSSA) had been carried out in NZ. Therefore, the presence of non-functional *mecC* in clinical MSSA isolates from other sources and from prior to 2021 cannot be ruled out.

The genomes obtained from the two *mecC*-PCR-positive isolates are in the same clonal complex and contain almost identical SCC*mec* elements with the same mutations in the *mecC* locus. The level of pairwise identity between the two isolates (99.95% ANI and 234 core genome SNPs) rules out the direct transmission or a very recent common ancestor between the two cases but is consistent with membership in a circulating lineage. Both cases resided in the South Island of NZ but lived approximately 250 km apart, and no epidemiological links between them could be determined. This suggests there may be undetected cases, or a reservoir, of *mecC*-positive *S. aureus* in livestock or wildlife populations.

The isolates sequenced in this study fall within CC49 and are the first clinical isolates from CC49 identified in NZ. On the core SNP phylogenetic tree, these isolates cluster with two isolates obtained from a single hedgehog sampled in NZ. European hedgehogs (*Erinaceus europaeus*) were introduced to NZ from the UK in the late nineteenth century. They are now widespread throughout the country and are common in both urban and rural areas. A survey of 59 hedgehogs in NZ in the 1960s found that 85% were infected with *S. aureus,* and of these, 86% exhibited resistance to penicillin [40]. Both cases in this study lived in rural areas and spent time gardening and may have come into contact with hedgehogs either directly or indirectly. However, conclusions on transmission between hedgehogs and humans cannot be drawn from only a single hedgehog, and we cannot rule out transmission from livestock, other animals or an environmental source.

Internationally, CC49 is rare in humans [41] and typically animal-associated, having been detected in a range of wildlife in Europe including wild boar, squirrels, rodents, voles and cats, in addition to hedgehogs [35,42]. CC49 has not been detected in studies of * S. aureus* in cattle in NZ to date [43,44], and its prevalence in other livestock or introduced mammals in NZ is unknown. In our isolates, we did not detect the *chp, sak*, or *scn* virulence factors, which is consistent with an animal origin for this lineage. These wSa3 phage-encoded virulence factors are normally present in human strains of *S. aureus*, where they are thought to be involved in modulating the innate immune response in humans [39], but are commonly absent in animal-derived *mecC*-positive strains [13].

Despite the overall close genomic relationship between the human and NZ hedgehog isolates in this study, there are considerable differences in the SCC*mec* elements between these isolates. The hedgehog isolates do not contain a full SCC*mec* type XI(8E) element, and their *mecC* gene is a different allotype (mecC2) more commonly seen in non-*aureus* staphylococcal and mammaliicoccal species. However, we only have sequence data from a single hedgehog from NZ in this study for comparison, so the prevalence of the SCC*mec* elements and genomic variability of *S. aureus* in hedgehogs in NZ is unknown. Isolates from CC49 are more commonly MSSA, with only 7 of the 61 isolates in our comparison containing a SCC*mec* type XI element (including only one hedgehog isolate). Phylogenetic comparisons of the SCC*mec* element in our human isolates and the other isolates in this study show it is more closely related to those from clonal complexes 130, 425 and 1943 than CC49. SCC*mec* type XI(8E) has been identified across Europe in a diversity of host species, including livestock, companion animals and wildlife [45], but has not previously been detected in NZ in either humans or animals [43,44]. Thus, it is unclear where the *mecC* cassette was acquired by our human isolates, and further sampling across livestock and wildlife populations will be required to determine its origin.

The *mecC* gene in our human isolates is truncated due to the presence of a deletion in the gene that introduces a premature stop codon. Consistent with this, both clinical cases in this study had an MSSA phenotype and were able to be treated successfully with beta-lactam antibiotics. We did not find any other instances in the literature or databases of *mecC* genes with similar deletions rendering them non-functional. However, it is possible that non-functional *mec* genes are not detected or reported in other studies where the presence or absence of methicillin resistance is only established by doing phenotypic susceptibility testing. In these cases, the MSSA phenotype may not be investigated further for the presence of *mec* genes. Reversion of *mecA* isolates from MRSA to MSSA phenotype has been occasionally observed in other studies where discrepancies between resistance phenotype and genotype are specifically investigated. Ledda *et al.* (2017) reported the re-emergence of an MSSA phenotype among ST36 *mecA*-positive *S. aureus* isolates [46]. For some of the isolates in their study, reversion to the MSSA phenotype was caused by the loss of the entire SCC*mec* cassette, but in one isolate, they observed a single bp deletion that caused a frameshift and premature stop codon in the *mecA* gene, similar to what we observed in the isolates in this study. In a study of 1470 strains of *S. aureus*, Kime *et al*. (2019) found that around 10% of the isolates had silenced resistance genes, including *mecA*, and in two isolates, antibiotic susceptibility was linked to frameshift mutations in the *mecA* gene [47]. Reversion from MRSA to MSSA may occur where there is little selection pressure to maintain the resistance phenotype, indicating that our isolates may have evolved in an environment where there is little or no antibiotic use. This lends weight to the hypothesis that the human infections were not the result of direct transmission from hedgehogs, as selection pressure from the *Trichophyton* fungus would be more likely to retain a functional *mecC* gene in *S. aureus* isolates from hedgehogs. However, as we do not know the origin of the SCC*mec* cassette in the CC49 strains reported here or at what point the cassette was acquired by these strains, we cannot determine at what point the *mecC* gene became non-functional.

In conclusion, our study characterizes two *S. aureus* isolates containing an SCC*mec* type XI element with a non-functional *mecC* gene in NZ, thus expanding the geographic range where *mecC S. aureus* is known to occur. These isolates are the first isolates from any species in NZ with a *mecC*-containing SCC*mec* type XI element and also the first human isolates from CC49 found in NZ. The presence of two cases with no epidemiological links indicates there may be a reservoir of *mecC S. aureus* in NZ. However, further sampling of livestock, wildlife and humans, particularly in the South Island, where the human cases were located, will be required to understand the prevalence of *mecC S. aureus* in NZ and ascertain the origin of the human infections.

## supplementary material

10.1099/acmi.0.000849.v2 Supplementary Material 1.

10.1099/acmi.0.000849.v2 Supplementary Material 2.

## Abbreviations

AMR: antimicrobial resistance
MRSA: methicillin-resistant *Staphylococcus aureus*
MSSA: methicillin-sensitive *Staphylococcus aureus*
ncRNA: non-coding RNA
NZ: New Zealand
SCC*mec*: staphylococcal cassette chromosome *mec*
UK: United Kingdom
WGS: whole-genome sequencing

## References

[1] Utsui Y, Yokota T (1985). Role of an altered penicillin-binding protein in methicillin- and cephem-resistant *Staphylococcus aureus*. Antimicrob Agents Chemother.

[2] Katayama Y, Ito T, Hiramatsu K (2000). A new class of genetic element, *Staphylococcus* cassette Chromosome *mec*, encodes methicillin resistance in *Staphylococcus aureus*. Antimicrob Agents Chemother.

[3] García-Álvarez L, Holden MTG, Lindsay H, Webb CR, Brown DFJ (2011). Meticillin-resistant *Staphylococcus aureus* with a novel mecA homologue in human and bovine populations in the UK and Denmark: a descriptive study. Lancet Infect Dis.

[4] Paterson GK, Harrison EM, Holmes MA (2014). The emergence of mecC methicillin-resistant *Staphylococcus aureus*. Trends Microbiol.

[5] Paterson GK, Larsen AR, Robb A, Edwards GE, Pennycott TW (2012). The newly described mecA homologue, mecALGA251, is present in methicillin-resistant *Staphylococcus aureus* isolates from a diverse range of host species. J Antimicrob Chemother.

[6] Vandendriessche S, Vanderhaeghen W, Soares FV, Hallin M, Catry B (2013). Prevalence, risk factors and genetic diversity of methicillin-resistant *Staphylococcus aureus* carried by humans and animals across livestock production sectors. J Antimicrob Chemother.

[7] Angen Ø, Stegger M, Larsen J, Lilje B, Kaya H (2017). Report of mecC-carrying MRSA in domestic swine. J Antimicrob Chemother.

[8] Worthing KA, Coombs GW, Pang S, Abraham S, Saputra S (2016). Isolation of mecC MRSA in Australia. J Antimicrob Chemother.

[9] Aklilu E, Chia HY (2020). First mecC and mecA positive livestock-associated methicillin resistant *Staphylococcus aureus* (mecC MRSA/LA-MRSA) from dairy cattle in Malaysia. *Microorganisms*.

[10] Silva JG, Araujo WJ, Leite EL, Dias LM, Vasconcelos PC (2021). First report of a livestock-associated methicillin-resistant *Staphylococcus aureus* ST126 harbouring the mecC variant in Brazil. Transbound Emerg Dis.

[11] Bengtsson B, Persson L, Ekström K, Unnerstad HE, Uhlhorn H (2017). High occurrence of mecC-MRSA in wild hedgehogs (*Erinaceus europaeus*) in Sweden. Vet Microbiol.

[12] Rasmussen SL, Larsen J, van Wijk RE, Jones OR, Berg TB (2019). European hedgehogs (*Erinaceus europaeus*) as a natural reservoir of methicillin-resistant *Staphylococcus aureus* carrying mecC in Denmark. PLoS One.

[13] Dierikx C, Hengeveld P, Witteveen S, van Hoek A, van Santen-Verheuvel M (2023). Genomic comparison of mecC-carrying methicillin-resistant *Staphylococcus aureus* from hedgehogs and humans in the Netherlands. J Antimicrob Chemother.

[14] Larsen J, Raisen CL, Ba X, Sadgrove NJ, Padilla-González GF (2022). Emergence of methicillin resistance predates the clinical use of antibiotics. Nature.

[15] Lozano C, Fernández-Fernández R, Ruiz-Ripa L, Gómez P, Zarazaga M (2020). Human mecC-Carrying MRSA: clinical implications and risk factors. *Microorganisms*.

[16] Petersen A, Stegger M, Heltberg O, Christensen J, Zeuthen A (2013). Epidemiology of methicillin-resistant *Staphylococcus aureus* carrying the novel mecC gene in Denmark corroborates a zoonotic reservoir with transmission to humans. Clin Microbiol Infect.

[17] Harrison EM, Paterson GK, Holden MTG, Larsen J, Stegger M (2013). Whole genome sequencing identifies zoonotic transmission of MRSA isolates with the novel mecA homologue mecC. EMBO Mol Med.

[18] Albert E, Sahin-Tóth J, Horváth A, Papp M, Biksi I (2023). Genomic evidence for direct transmission of *mecC*-MRSA between a horse and its veterinarian. Antibiotics.

[19] Pichon B, Hill R, Laurent F, Larsen AR, Skov RL (2012). Development of a real-time quadruplex PCR assay for simultaneous detection of nuc, Panton-Valentine Leucocidin (PVL), mecA and homologue mecALGA251. J Antimicrob Chemother.

[20] Strommenger B, Braulke C, Heuck D, Schmidt C, Pasemann B (2008). spa typing of *Staphylococcus aureus* as a frontline tool in epidemiological typing. J Clin Microbiol.

[21] Chen S, Zhou Y, Chen Y, Gu J (2018). fastp: an ultra-fast all-in-one FASTQ preprocessor. Bioinformatics.

[22] Kim D, Song L, Breitwieser FP, Salzberg SL (2016). Centrifuge: rapid and sensitive classification of metagenomic sequences. Genome Res.

[23] Souvorov A, Agarwala R, Lipman DJ (2018). SKESA: strategic k-mer extension for scrupulous assemblies. Genome Biol.

[24] Gurevich A, Saveliev V, Vyahhi N, Tesler G (2013). QUAST: quality assessment tool for genome assemblies. Bioinformatics.

[25] Kolmogorov M, Yuan J, Lin Y, Pevzner PA (2019). Assembly of long, error-prone reads using repeat graphs. Nat Biotechnol.

[26] Walker BJ, Abeel T, Shea T, Priest M, Abouelliel A (2014). Pilon: an integrated tool for comprehensive microbial variant detection and genome assembly improvement. PLoS One.

[27] Hunt M, Otto TD, Parkhill J, Keane JA, Silva (2015). Circlator: automated circularization of genome assemblies using long sequencing reads. Genome Biol.

[28] Schwengers O, Jelonek L, Dieckmann MA, Beyvers S, Blom J (2021). Bakta: rapid and standardized annotation of bacterial genomes via alignment-free sequence identification. Microb Genom.

[29] Jolley KA, Maiden MCJ (2010). BIGSdb: scalable analysis of bacterial genome variation at the population level. BMC Bioinform.

[30] Jain C, Rodriguez-R LM, Phillippy AM, Konstantinidis KT, Aluru S (2018). High throughput ANI analysis of 90K prokaryotic genomes reveals clear species boundaries. Nat Commun.

[31] Feldgarden M, Brover V, Gonzalez-Escalona N, Frye JG, Haendiges J (2021). AMRFinderPlus and the reference gene catalog facilitate examination of the genomic links among antimicrobial resistance, stress response, and virulence. Sci Rep.

[32] Kaya H, Hasman H, Larsen J, Stegger M, Johannesen TB (2018). SCC*mec*Finder, a web-based tool for typing of Staphylococcal cassette chromosome *mec*in *Staphylococcus aureus* using whole-genome sequence data. mSphere.

[33] Nguyen L-T, Schmidt HA, von Haeseler A, Minh BQ (2015). IQ-TREE: a fast and effective stochastic algorithm for estimating maximum-likelihood phylogenies. Mol Biol Evol.

[34] Stamatakis A (2014). RAxML version 8: a tool for phylogenetic analysis and post-analysis of large phylogenies. Bioinformatics.

[35] Martínez-Seijas C, Mascarós P, Lizana V, Martí-Marco A, Arnau-Bonachera A (2023). Genomic characterization of *Staphylococcus aureus* in wildlife. Animals.

[36] Walsh SK, Imrie RM, Matuszewska M, Paterson GK, Weinert LA (2023). The host phylogeny determines viral infectivity and replication across *Staphylococcus* host species. PLoS Pathog.

[37] Davis R, Hossain MJ, Liles MR, Panizzi P (2013). Complete genome sequence of *Staphylococcus aureus* tager 104, a sequence type 49 ancestor. Genome Announc.

[38] Davis RW, Brannen AD, Hossain MJ, Monsma S, Bock PE (2016). Complete genome of *Staphylococcus aureus* Tager 104 provides evidence of its relation to modern systemic hospital-acquired strains. BMC Genom.

[39] Howden BP, Giulieri SG, Wong Fok Lung T, Baines SL, Sharkey LK (2023). *Staphylococcus aureus* host interactions and adaptation. Nat Rev Microbiol.

[40] Smith JM (1965). *Staphylococcus aureus* strains associated with the hedgehog, *Erinaceus europaeus*. Epidemiol Infect.

[41] Monecke S, Gavier-Widén D, Hotzel H, Peters M, Guenther S (2016). Diversity of *Staphylococcus aureus* isolates in European wildlife. PLoS One.

[42] Silva V, Capelo JL, Igrejas G, Poeta P (2020). Molecular epidemiology of *Staphylococcus aureus* lineages in wild animals in Europe: a review. Antibiotics.

[43] Greening SS, Zhang J, Midwinter AC, Wilkinson DA, McDougall S (2021). The genetic relatedness and antimicrobial resistance patterns of Mastitis-causing *Staphylococcus aureus* strains isolated from new Zealand Dairy Cattle. Vet Sci.

[44] Nesaraj J, Grinberg A, Laven R, Biggs P (2023). Genomic epidemiology of bovine mastitis-causing *Staphylococcus aureus* in New Zealand. Vet Microbiol.

[45] Liu J, Chen D, Peters BM, Li L, Li B (2016). Staphylococcal chromosomal cassettes mec (SCCmec): a mobile genetic element in methicillin-resistant *Staphylococcus aureus*. Microb Pathog.

[46] Ledda A, Price JR, Cole K, Llewelyn MJ, Kearns AM (2017). Re-emergence of methicillin susceptibility in a resistant lineage of *Staphylococcus aureus*. J Antimicrob Chemother.

[47] Kime L, Randall CP, Banda FI, Coll F, Wright J (2019). Transient silencing of antibiotic resistance by mutation represents a significant potential source of unanticipated therapeutic failure. mBio.

