# Supplementary material for: Genomic epidemiology of mecC-carrying Staphylococcus aureus isolates from human clinical cases in New Zealand
Source: Access Microbiol. 2024 Sep 5;6(9):000849.v2. doi: 10.1099/acmi.0.000849.v2 (PMC11376224; doi:10.1099/acmi.0.000849.v2)
Supplement: Supplementary Material 2. [file acmi-6-00849-s002.pdf]

Supplementary Figure 1

RAxML-generated phylogeny of SCCmec elements from Isolates 1 and 2 (this study), and *mecC*-MRSA isolates from CC49, CC130, CC425, CC1943, CC2616 and CC599. Tree tips are coloured according to their CC type.

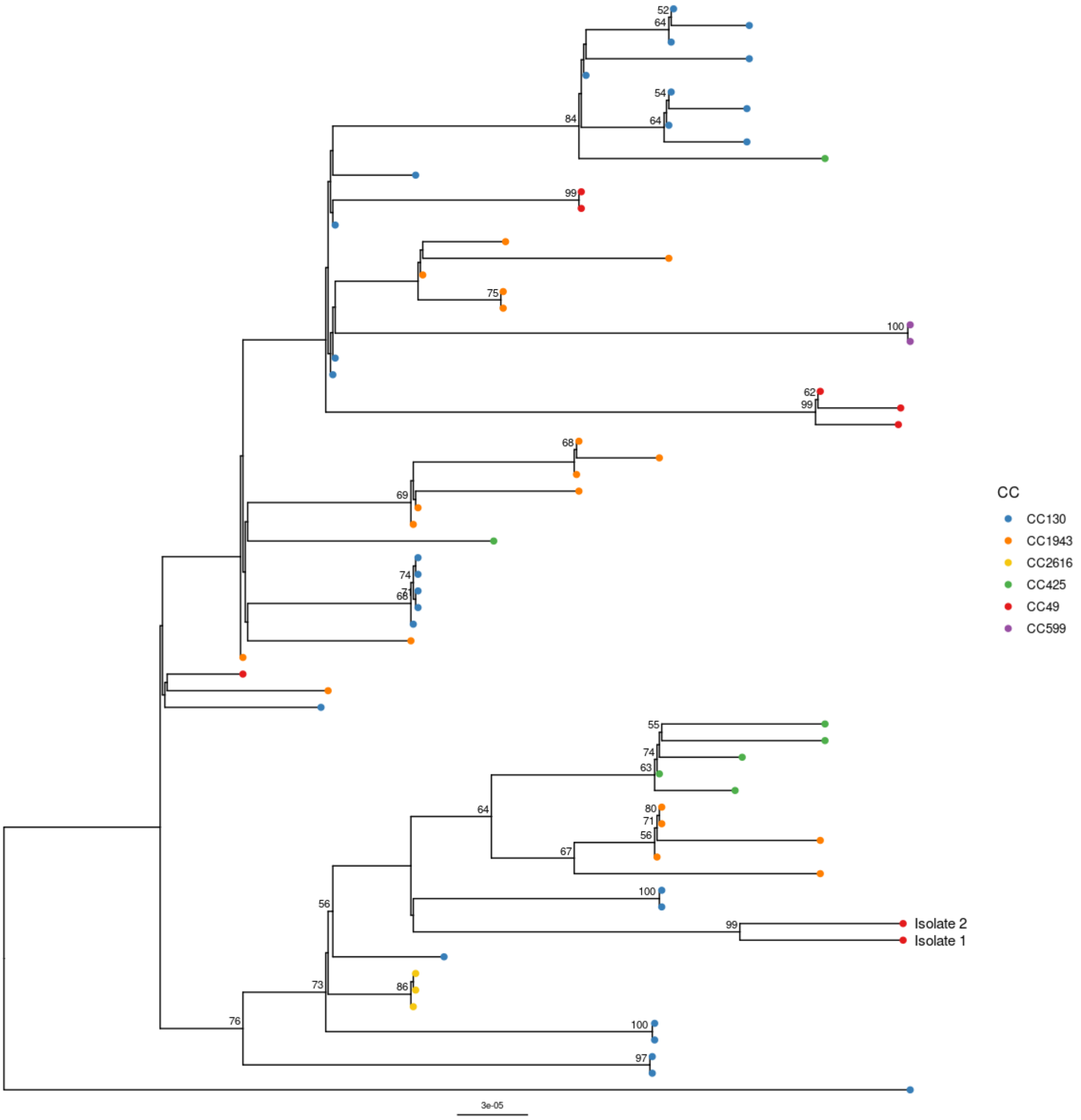

Supplementary Table 1

See Excel file submitted separately

Supplementary Table 2

Resistance, virulence and stress genes identified by AMRfinder. Note that *mecC* was not detected due to the presence of a frameshift mutation.

	22MR0019	22MR1788
**AMR**	blaZ, mecI, mepA, tet(38)	blaZ, mecI, mepA, tet(38)
**STRESS**	arsB, arsC, lmrS	arsB, arsC, lmrS
**VIRULENCE**	aur, hld, hlgA, hlgB, hlgC, icaC, lukE, sel26, selX, splA, splB, splE,	aur, hld, hlgA, hlgB, hlgC, icaC, lukE, sel26, selX, splA, splB, splE,

CONFIDENTIAL
